# Diagnostic Dilemma of an Imperforate Hymen: A Rare Case With Atypical Symptoms

**DOI:** 10.7759/cureus.83179

**Published:** 2025-04-29

**Authors:** Petra G Parikesit, Devanya T Kirani, Caga D Triroso, Theresia Avilla R Kusumosih

**Affiliations:** 1 Department of Obstetrics and Gynaecology, Duta Wacana Christian University, Yogyakarta, IDN; 2 Department of Obstetrics and Gynaecology, Bethesda Hospital, Yogyakarta, IDN

**Keywords:** adolescent gynecology, hematocolpometra, hymenectomy, imperforate hymen, primary amenorrhea

## Abstract

An imperforate hymen (IH) is an uncommon congenital condition resulting from the failure of the synovaginal bulbus to canalize during embryonic development. While it typically presents with primary amenorrhea, cyclic pain, and hematocolpos, certain cases may exhibit unusual symptoms like constipation and obstructive uropathic disorders, which can often result in a delayed diagnosis. A 12-year-old female adolescent was observed with a history of progressively worsening cyclic abdominal pain, along with severe constipation and mild urinary disturbances. The physical examination revealed a suprapubic mass, as well as a hymen that entirely obstructed the vaginal introitus, showing a bluish protrusion. Initially, the patient received a diagnosis of vaginal agenesis; however, further examination revealed the presence of hematocolpometra linked to IH. The patient received a hymenectomy, resulting in symptomatic improvement and full resolution of the hematocolpos. The infrequency and clinical diversity of IH highlight the critical need for early detection via genital examination from birth, particularly to prevent misdiagnosis that could postpone treatment. Imaging techniques like ultrasound are crucial for distinguishing IH from related conditions, including vaginal agenesis. Additional investigations are essential to enhance clinical understanding and guarantee effective management for individuals affected by this condition.

## Introduction

An imperforate hymen (IH) is an uncommon condition characterized by the complete obstruction of the vaginal lumen by a thin membrane, which typically possesses a small opening. The prevalence is uncommon, affecting approximately one in 1,000 to 10,000 women, translating to a rate of 0.05%-0.1% globally [1]. The inability to canalize the synovaginal bulbus, at the junction of the Müllerian duct and the urogenital sinus, is the underlying cause of IH. The symptoms of IH typically manifest at menarche, characterized by cyclic pelvic or lower abdominal pain, primary amenorrhea, a bluish protrusion in the perineum, and urinary retention [2]. A comprehensive evaluation is essential and includes inspection of the vagina and surrounding area, abdominal and pelvic palpation, and rectal examination. This assessment aims to identify the presence of menstrual blood accumulation in the vagina or uterus (hematocolpometra). Should the pelvic mass subsequently manifest as a notable, blue perineal mass upon physical examination, it is classified as hematometra or hematocolpos. In the absence of urgent complications, such as renal impairment, infection, or risk of infertility, deferring hymenectomy until adolescence is recommended, as this allows for estrogenization of the hymenal tissue, which facilitates improved surgical outcomes, reduces the risk of scarring and postoperative infection, and accounts for the possibility of spontaneous hymenal perforation during this developmental stage [3]. Nevertheless, the hymenectomy procedure, along with the drainage of accumulated blood, is considered the definitive intervention to prevent complications associated with IH, including subfertility, endometriosis, hydronephrosis, and renal failure [4]. This report seeks to elucidate the atypical clinical manifestation of a case of IH, with the intention of offering insights into the diagnostic complexities inherent to this rare condition.

## Case presentation

A 12-year-old female patient presented to the referral hospital with a complaint of abdominal pain described as a wrapping sensation, which had been recurring for the past two months. At first, the pain did not impact the patient's quality of life. In the past week, the intensity of the pain has been such that it has significantly hindered the ability to engage in daily activities. The patient also complained of not defecating for five days, followed by the presence of blood during defecation on the sixth day. The patient reported experiencing mild discomfort during urination. The patient presented with a subfebrile temperature (approximately 37.0°C to 37.4°C) for two days prior to hospital admission, accompanied by a single episode of nausea and vomiting. The patient has not yet attained menarche; however, the onset of thelarche and pubarche is evident. The patient admitted that she had not been sexually active, with no documented family history of gynecological disorders. Additionally, there was no record of antibiotic or cytotoxic drug use, nor any radiation exposure during the course of her mother's pregnancy. She was delivered spontaneously, exhibiting weight and length consistent with the gestational age.

**Figure 1:**
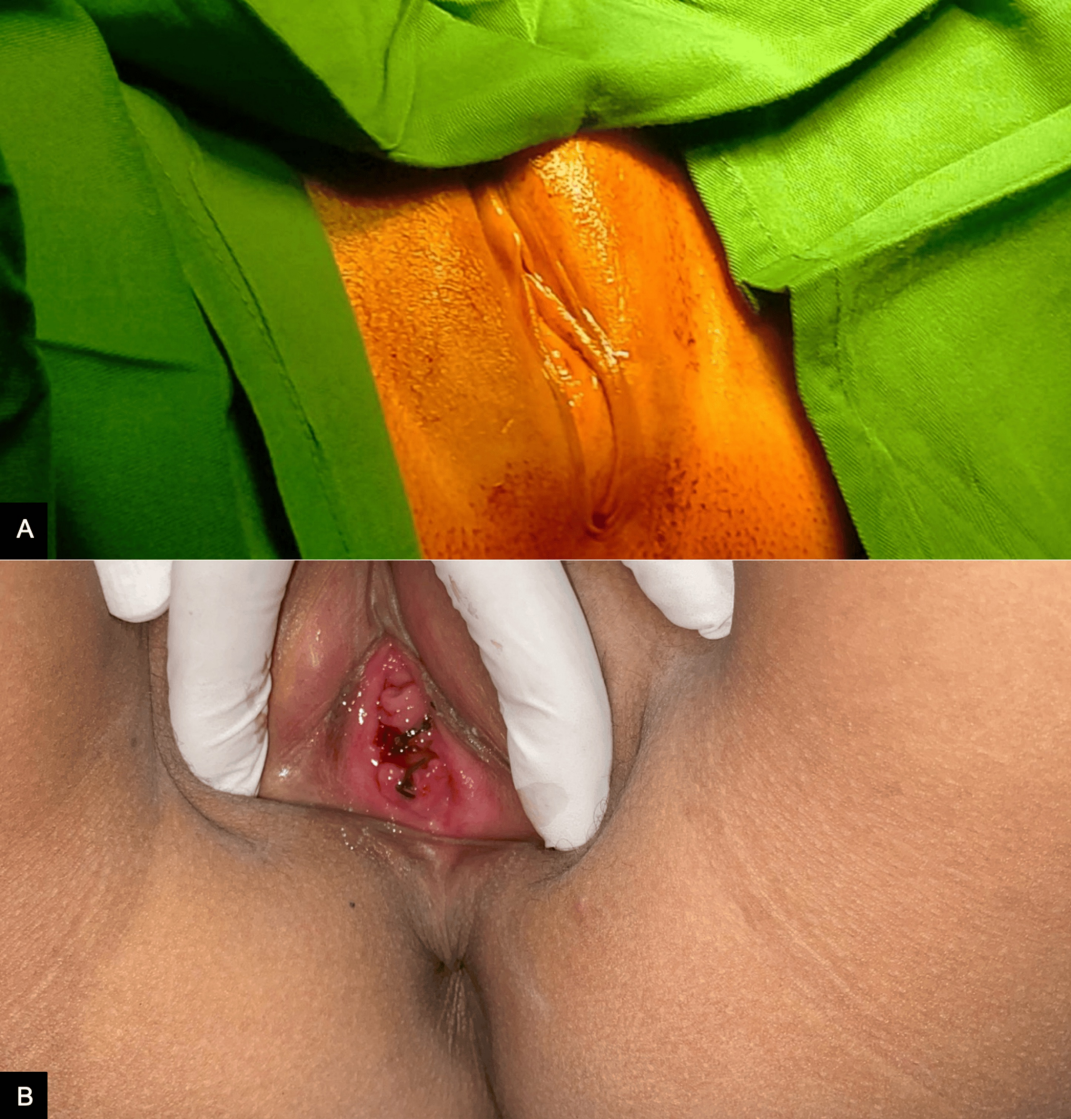
An imperforate hymen presents as a bulging, bluish discoloration (A) and observations following hymenectomy performed with a cruciate incision (B).

The patient's vital signs were within normal limits, with the exception of a pulse rate of 126 beats per minute (bpm). During the subsequent physical examination, a well-defined, tender, non-mobile suprapubic mass with a cystic consistency, measuring 5×6 cm, was palpated approximately one fingerbreadth below the umbilicus. Upon examination of the external genitalia, a hymen was observed covering the entire vaginal introitus, exhibiting a bulging purplish-blue coloration. No vaginal ostium was identified, and the area was noted to be painful to touch, without blood or discharge. The labia minora and majora appeared normal (Figure 1A).

**Figure 2:**
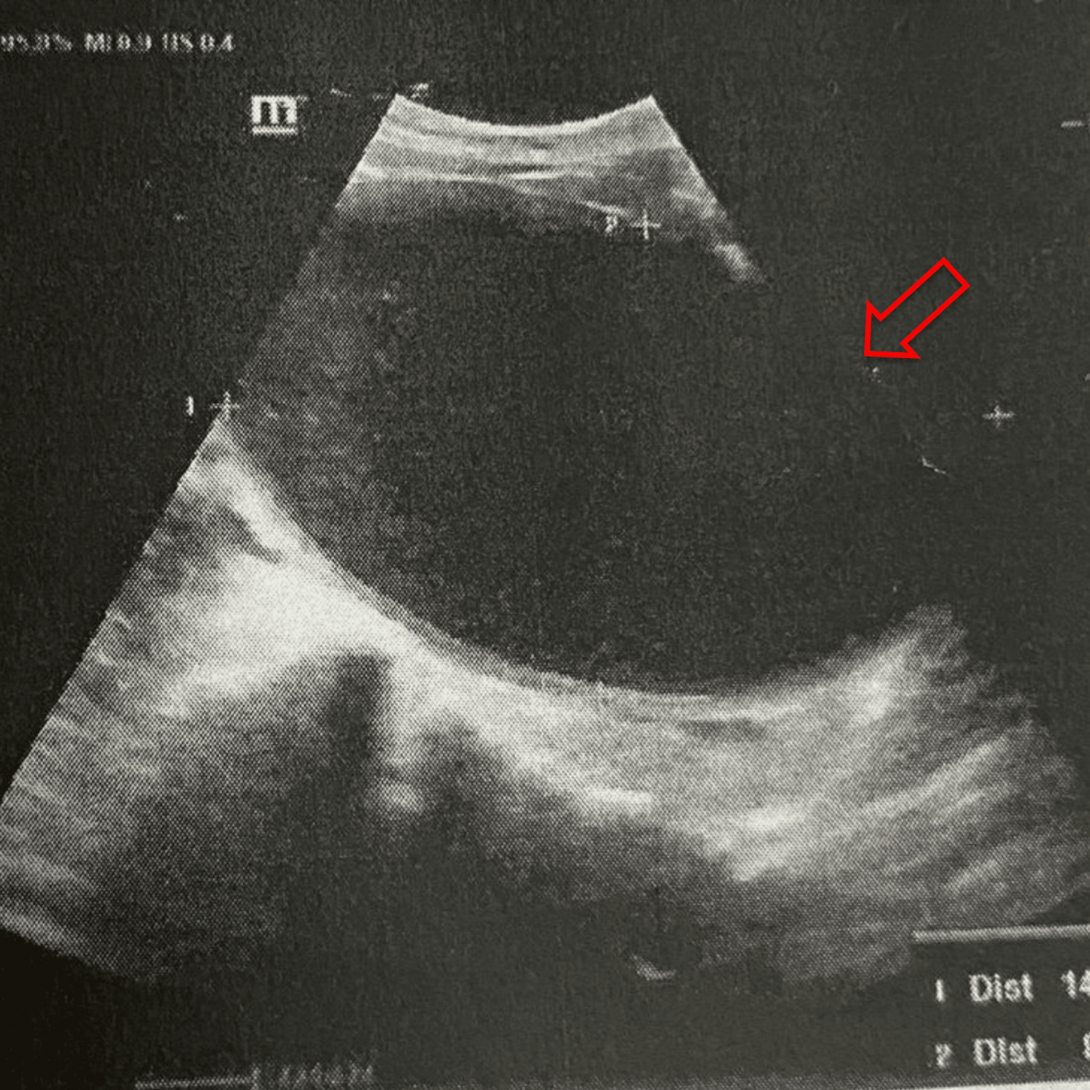
Transabdominal ultrasound shows an anechoic cystic mass at the right adnexa (red arrow).

**Figure 3:**
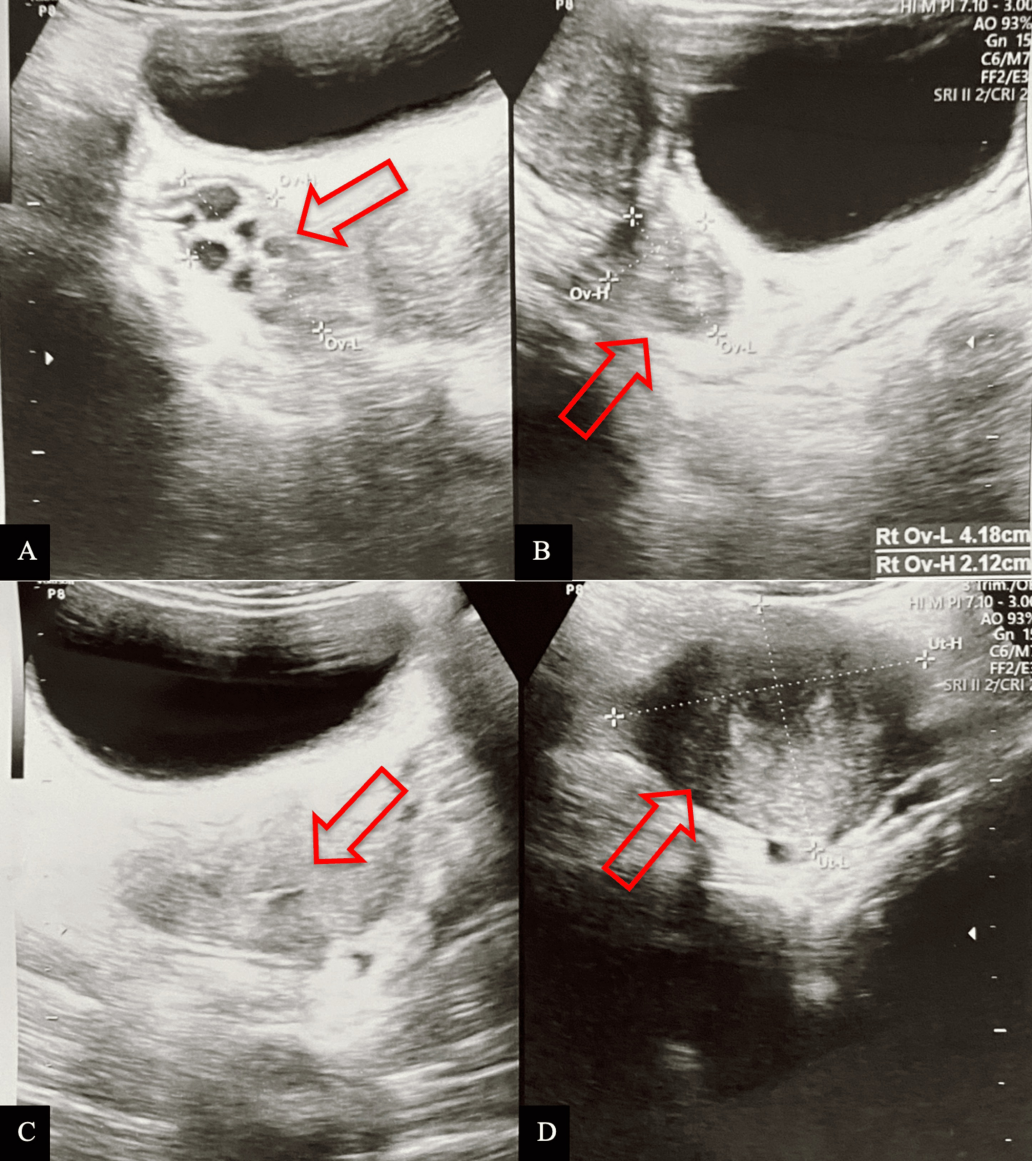
Transabdominal ultrasound shows a polycystic structure suggestive of residual blood on the left ovary (A), a normal right ovary (B), an empty canalis vaginalis (C), and an empty uterine cavity (D) shown by a red arrow.

The initial ultrasound examination revealed an impression of hematocolpos, indicating a potential case of vaginal agenesis. Subsequently, a follow-up ultrasound assessment at the referral facility revealed a solitary rounded anechoic cystic mass located at the right adnexa, measuring 12x9x8 cm, indicating the potential diagnosis of hematocolpometra secondary to an imperforate hymen. The intra-abdominal assessment revealed an absence of free fluid, and the bilateral kidneys were observed to be within normal limits (Figure 2). The patient received ketorolac 3x30mg for pain management, in addition to a preoperative dose of cefazolin 2 grams, and was scheduled for hymen surgery the next day. During the examination under spinal anesthesia, an obstruction of the vaginal canal at the introitus was confirmed, aligning with the preoperative diagnosis of imperforate hymen. During the cross-incision hymenectomy, approximately 1000 cc of dark red blood was evacuated through the incision site, resulting in a noticeable reduction in the size of the abdominal mass. Following the incision, the surgical site was irrigated with 0.9% normal saline solution, and a circumferential marsupialization suture was subsequently performed. Several hours following the surgical procedure, the patient experienced mild discomfort at the surgical site and was able to defecate without the presence of blood. The patient was discharged with a prescription for mefenamic acid and advised to take 500 mg three times daily for pain management. At the one-week postoperative follow-up, the suprapubic mass was entirely nonpalpable, defecation issues had resolved, pain levels had markedly diminished, and there was notable improvement in the surgical wound (Figure 1B). Ultrasound findings revealed an empty vaginal and uterine cavity, with normal morphology of the right ovary. However, the left ovary demonstrated multiple cystic formations, likely representing residual hemorrhagic changes secondary to recent hematocolpometra. These findings are consistent with a post-obstructive ovarian response and early resolution following surgical decompression (Figures 3A-3D).

## Discussion

This report presents an uncommon instance of IH, a congenital anomaly affecting the female reproductive system, identified in a 12-year-old female patient. Based on current understanding, IH typically presents with characteristic symptoms including primary amenorrhea, a palpable pelvic mass, or urine retention/obstructive uropathy resulting from hematocolpos [3]. In this instance, the patient exhibited a primary complaint of primary amenorrhea, coupled with significant constipation and discomfort during urination, rendering her clinical presentation both atypical and worthy of further examination. The diagnosis of IH, particularly in the presence of atypical complications, presents considerable challenges, especially when associated with an insufficient clinical evaluation.

The diagnosis of IH frequently experiences delays, as the condition is typically asymptomatic in its early stages until there is an accumulation of menstrual blood, which is accompanied by less specific symptoms. IH is typically identified at the onset of menstruation, when the accumulation of blood in the vagina exerts pressure on the bladder or urethra, resulting in discomfort and painful urination. The diagnosis of IH prior to the onset of puberty presents challenges, as this condition is typically identified following the initiation of pubertal development. Early diagnosis of IH can occur if a gynecological examination is conducted on a newborn or if there are indications such as a palpable abdominal mass, which may present as hydrocolpos or mucocolpos. In pediatric patients, conducting a hymen examination is crucial, focusing on the morphology of the hymen, variations in the hymenal margin, and any indications of trauma or scarring. Through a gynecological examination of the newborn, IH can be identified at an early stage [5]. IH frequently remains undiagnosed owing to the non-specific nature of history taking and insufficient physical examination. Consequently, IH is typically identified only when symptoms manifest and subsequent diagnostic evaluations, such as ultrasound, are conducted [6].

The patient presented with a one-week history of increased constipation, accompanied by mild urinary symptoms. In instances of IH, the occurrence of constipation is infrequent; however, it may arise as a result of pressure from hematocolpos or hematometra on the rectum. This condition can necessitate a significant volume of blood, as demonstrated in our case, where 1000 cc of blood was identified during evacuation [7]. We propose that the severe constipation, alongside the mild urinary symptoms, represents an atypical manifestation of IH. These nonspecific symptoms could potentially lead to a misdiagnosis, especially since the combination of constipation and a suprapubic mass can mimic bowel obstruction. Therefore, it is crucial to identify specific symptoms indicative of IH, such as primary amenorrhea.

Atypical clinical manifestations in IH cases can mimic vaginal agenesis, often obscuring the diagnosis. In our case, the patient had manifestations of primary amenorrhea with cyclic pain, no vaginal ostium, and normal age-appropriate pubertal growth and development. These manifestations can also be found in patients with vaginal agenesis. However, if examined further in IH, the failure of the inferior end of the vaginal plate to form a canal causes blood to tend to accumulate in the vagina so that it will appear as a bluish protrusion of the hymen [1]. Meanwhile, in the case of vaginal agenesis, the condition is generally caused by failure of development of the paired Müllerian ducts, which is clinically characterized by a shallow vaginal dimple without a true vaginal canal and a hypoplastic or incompletely developed uterus [8].

In order to assist with the diagnosis, a suprapubic ultrasound examination may be deemed appropriate given its accessibility and cost-effectiveness for identifying blood accumulation in the vagina (hematocolpos) or a small, dilated uterine cavity resulting from hematometra, which could progress to a hematosalphinx if not addressed. In advanced instances, compression of the urinary tract due to hematocolpos may result in ureteropelvic dilatation and hydronephrosis [6]. The conclusive approach for managing instances of IH involves surgical intervention through hymenectomy. A cruciate incision or excision is executed on the hymen to establish an opening within the hymenal structure. Following the incision, the trapped blood will be expelled through drainage, and irrigation will be performed. The incised tissue is meticulously sutured to the adjacent tissue to create a hymenal burrow [9]. Irrigation serves to reduce the risk of surgical site infections by removing debris, blood, and microbial contaminants from the operative field while maintaining tissue hydration to support healing and facilitate intraoperative assessment. Additionally, it enhances visualization of viable structures by clearing nonviable tissue, thereby aiding in surgical precision [10]. At our hospital, the patient was managed with a cruciate incision technique, followed by thorough irrigation of the operative field and placement of circumferential marsupialization sutures. This approach led to significant clinical improvement in the patient's postoperative condition.

## Conclusions

An IH presents a diagnostic challenge owing to its infrequency and diverse clinical presentations. While typically defined by primary amenorrhea, cyclic abdominal pain, and hematocolpos, certain cases may additionally exhibit symptoms such as constipation and obstructive uropathy, frequently resulting in misdiagnosis. Timely identification via physical assessment of the neonate and imaging techniques is crucial to avert additional complications.
